# Tobacco use amongst out of school adolescents in a Local Government Area in Nigeria

**DOI:** 10.1186/1747-597X-5-24

**Published:** 2010-10-18

**Authors:** Akindele O Adebiyi, Babalola Faseru, Adesola O Sangowawa, Eme T Owoaje

**Affiliations:** 1 Department of Community Medicine, College of Medicine, University College Hospital, Ibadan, Nigeria; 2 University of Kansas Medical Centre and School of Medicine, Kansas City, Kansas, USA; 3 Institute of Child Health, College of Medicine, University of Ibadan, Nigeria

## Abstract

**Introduction:**

Out-of-school adolescents are often neglected when planning for tobacco prevention programmes whereas they are more vulnerable. Few studies exist in Nigeria about their pattern of tobacco use to serve as the basis for effective policy formulation.

**Method:**

A sub sample of 215 out of school adolescents was analyzed from a descriptive cross sectional study on psychoactive substance use amongst youths in two communities in a Local Government Area in Nigeria which used a multi-stage sampling technique.

**Results:**

Males were 53% and females 47%. Only 20.5% had ever used tobacco while 11.6% were current users. Males accounted for 60% of current users compared to 40% amongst females. Of current users, 84% believed that tobacco is not harmful to health. In addition, the two important sources of introduction to tobacco use were friends 72% and relatives 20%. Use of tobacco amongst significant others were: friends 27%, fathers 8.0%, relatives 4.2% and mothers 0.5%. The most common sources of supply were motor parks 52% and friends 16%.

**Conclusion:**

The study showed that peer influence is an important source of introduction to tobacco use while selling of tobacco to adolescents in youth aggregation areas is common. We advocate for a theory based approach to designing an appropriate health education intervention targeted at assisting adolescents in appreciating the harmful nature of tobacco use in this locality. A point-of-sale restriction to prevent adolescent access to tobacco in youth aggregation areas within the context of a comprehensive tobacco control policy is also suggested. However, more research would be needed for an in-depth understanding of the tobacco use vulnerability of this group of adolescents.

## Background

Tobacco use has become a rapidly growing problem worldwide as well as in many developing countries. It is projected that over the next 50 years close to 450 million deaths would be caused by tobacco use [1]. While it has been established that many smokers start before the age of 18 years, [2] of serious concern, is the increasing trend in smoking prevalence amongst youths and the likelihood that many of these young people who begin to smoke at an early age, will continue to do so throughout adulthood [3]. Furthermore, the years of potential life lost attributable to tobacco related diseases will continue to increase if we do not target interventions to prevent smoking initiation among youths. Factors associated with increasing uptake of smoking behavior among youths include low self-esteem, stressful life events, friends who smoke, advertisement and living with a smoker [4-6].

The patterns of tobacco use however vary from region to region and interventions for tobacco control also differ. In developed countries, systematic data collection procedures are available for documenting the prevalence and pattern of tobacco use but these procedures are sub-optimal in developing countries [7]. Hence, there is lack of adequate research to guide policy and interventions. Although studies examining smoking among youths have been documented in Nigeria, these are skewed towards describing pattern of use amongst in-school youths in urban areas [4,6,8]. However, many of the factors associated with adverse health behaviors which may include smoking initiation and persistence are known to be commoner amongst out-of-school youth because of their aggregation in areas lacking adult supervision. The term "out-of-school youth" is used to define several groups of young people: those who have dropped out of school, those who never attended school, or those who participate in non formal school programs [9]. These youths are a diverse group who may have completed elementary school (but lack basic skills to progress to high school or vocational training), dropped out or never started school [9]. Those who drop out of school may fail to acquire fundamentals of basic education and life skills [10]. This study describes tobacco use amongst a set of adolescents who are more likely to be overlooked by program planners [9]. Presently, this group is under-studied and under-represented in smoking prevention interventions in Nigeria. Generally speaking, literature is lacking on the availability of a sound theoretical basis for developing interventions aimed at adolescent smoking cessation [11]. It is thus hoped that this study would provide a template for the derivation of a theory-based approach to researching and understanding adolescent tobacco use as the basis for effective policy formulations.

## Methodology

The descriptive cross sectional study which is nested in a study of psychoactive substance use amongst youths was conducted in Kajola Local Government Area (LGA) of Oyo State in southwest Nigeria. We describe herein tobacco use amongst out-of-school adolescents. The LGA has six towns and one hundred and seventeen villages. Multi-stage sampling technique was used to select the study population of adolescents aged 10 - 19 years found in youth aggregation areas like garages and market places during normal school hours. Although the universe of the out-of-school adolescent is difficult to define; nevertheless many more adolescents drop out of school in rural (14.1%) and urban (7.5%) in the sixth grade in Nigeria (final class in primary school) and this roughly corresponds to around the age of 10-12 years averagely [12]. Usually, a youth of over 19 years is not expected to still be in secondary school. Thus, we recruited only adolescents aged 10 - 19 years for this study. Two towns - Okeho and Ilero with populations of approximately 48,817 and 52,613 respectively were selected by simple random sampling from the three transitional towns (i.e., rural towns' slowly undergoing urbanization). The youth aggregation areas were treated as clusters and six aggregation points were selected by simple random sampling from the identified seven points. The leaders of these aggregation points i.e., motor park union and day market leaders were consulted and gave their consent for the adolescents within their domains to be recruited for the study. The natural group leaders of the adolescents were also consulted and the purpose of the study explained to them after which they were enlisted to assist in the recruitment of all willing adolescents in these aggregation areas. We obtained verbal informed consent from every participating adolescent who was over 18 years and documented same on the questionnaire. Those who were minors gave their assent after the same procedure of explaining what the study entailed and the benefits derivable from it. All consenting and assenting adolescents found in these aggregation areas were included in the original study on psychoactive substance among adolescents on and around the streets during normal schooling hours. In this report, we only consider for analysis all consenting and assenting out-of-school adolescents satisfying the inclusion criteria of not currently schooling or having dropped out of school, never started school or having informal education classes of less than thrice per week. All presently schooling adolescents who were either playing truancy by not being in school or doing part-time job around these aggregation points were excluded from this analysis. The questionnaire was developed from a review of literature on psychoactive substance use in Nigeria and the section on tobacco use contained questions on tobacco use and reasons for use, source(s) of introduction to use and sources of regular use, perception of harm due to tobacco and tobacco use by significant others (i.e., those close to the respondents that may have a tremendous influence on respondents). Tobacco use was taken as referring to both cigarette and snuff use. Ever use was taken as tobacco use at least once before and current use taken as tobacco use within the last 30 days. The questionnaire was translated to the native language of the area (Yoruba) and back translated to English. It was then pre-tested subsequently amongst adolescents in similar settings in one of the wards in Okeho not used for the study. Ambiguous questions and errors in the questionnaires were corrected. Ethical approval was obtained from the Joint University of Ibadan/University College Hospital ethical review board while permission for the study was granted by the Primary Health Department of Kajola Local Government and local trade union leaders in the study area. Two hundred and fifteen out-of-school adolescents were enrolled.

## Results

The majority of respondents were within the 15-19 years age groups while males accounted for a little over half of the respondents. Almost all (93.5%) were single/unmarried. Close to two-thirds (64.2%) of the respondents have multiple and unstable sources of income. Conversely, hawking (13.5%) and apprenticeship (7.9%) were the prominent stable occupation of respondents. All these are shown in table 1.

**Table 1:** Socio-demographic characteristics of 215 out-of-school adolescents surveyed

Variable	n	%
***Age***		

10-14 years	25	11.6
15-19 years	190	88.4

***Employment status***		

Hawking	29	13.5
Apprenticeship	17	7.9
Driving	12	5.6
Car washing	11	5.1
Artisan	8	3.7
*Unstable employment/unemployed	138	64.2

***Sex***		

Male	114	53.0
Female	101	47.0

***Marital status***		

Single	201	93.5
Married	7	3.3
Cohabiting	7	3.3

*These had no particular stable employment/are employed for a day or two in a week/are unemployed

Table 2 shows that forty four (20.5%) of the youths have ever used tobacco at least once and 25 (11.6%) of the respondents currently use tobacco. Males accounted for 68.2% and 60.0% of ever and current users respectively.

**Table 2:** Tobacco use characteristics

Variable	n	%
***Ever use of tobacco***	***N = 215***	

Yes	44	20.5
No	171	79.5

***Current use of tobacco***	***N = 215***	

Yes	25	11.6
No	190	88.4

***Ever users***	***N = 44***	

Females	14	31.8
Males	30	68.2

***Current users***	***N = 25***	

Female	10	40.0
Male	15	60.0

**Figure 1:**
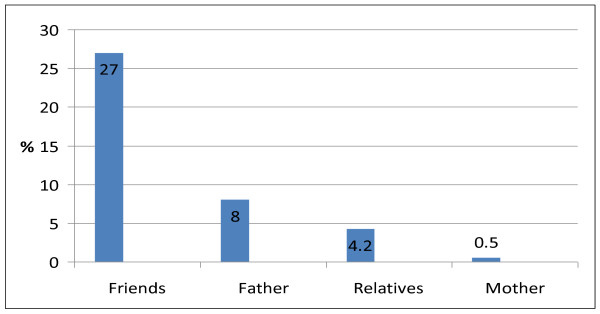
**Tobacco use amongst significant others (n = 215)**.

**Figure 2:**
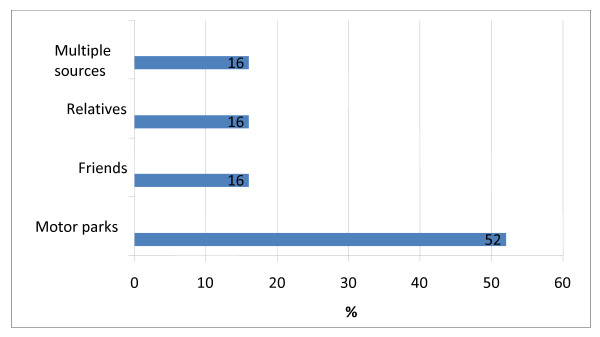
**Sources of current tobacco use (n = 25)**.

As presented in table 3, majority (72%) of current users were introduced to tobacco use by friends followed by relatives in 20% of cases. The prominent reasons given for ever use of tobacco included that it makes them high and bold (36.4%), their friends are using it (22.7%) and that they use it to sleep (13.6%). The prominent reasons for current use was not too different and included: to feel high and bold (44%), their friends are also using it (16%) and to feel cool (16%). Figure 1 shows that amongst significant others, tobacco use was highest amongst friends 58 (27%) followed by fathers 17 (8%) and relatives 9 (4.2%). Only one respondent reported tobacco use by her mother. Majority (52%) of current users of tobacco had retail stores in the motor parks as their regular source of purchase, while friends and relatives were the regular source in 16% of cases each. This is captured in Figure 2.

**Table 3:** Introduction to tobacco use and reasons for its use

Variable	n	%
**Introduction to tobacco use (N = 25)**		

Friends	18	72
Relatives	5	20
Casual contacts	2	8

**Reasons for tobacco use**		

***Ever use (N = 44)***		
To feel high and bold	16	36.4
My friends are using it	10	22.7
To sleep	6	13.6
*Others	12	27.3

***Current use (N = 25)***		

To feel high and bold	11	44.0
My friends are using it	4	16.0
To stay cool	4	16.0
**Others	6	24.0

*To keep awake, to stay cool, to lessen hunger, to take pain away

**Takes my pain away, to sleep, to stay awake

Overall as reflected in table 4, eighty seven (40.5%) of respondents believed that tobacco use was not harmful to health. Furthermore, 21 (84%) current tobacco users believed that tobacco use was not harmful to health.

**Table 4:** Perception about tobacco use

Perception	n	%
**All respondents (N = 215)**		

Believed tobacco use is harmful to health	128	59.5

Believed tobacco use is not harmful to health	87	40.5

**Current tobacco users (N = 25)**		

Believed tobacco use is harmful to health	4	16.0

Believed tobacco use is not harmful to health	21	84.0

## Discussion

Adolescence is a period of identity forming and great pressure. As this period presents an opportunity for picking up bad habits, it also presents a golden opportunity for behavior modification. However, out-of-school youths are especially vulnerable to wrong information from sources that lack credibility [9]. Because they are outside a formal school system they also miss out of the opportunity for learning in conducive environments.

One-fifth of the respondents have ever used tobacco. Conversely among secondary school students in Oyo State, Nigeria, Yisa and co-workers reported a lifetime prevalence of 10.6% [13]. Out-of-school adolescents sometimes go through many stressful life events compared to their in-school counterparts. For instance, in this current study, up to 60% of these youths have non-stable sources of income. Stressful life events have been shown to be associated with increased risk for smoking and alcohol use [5].

Not surprisingly, a smaller fraction of those who had ever used tobacco were self reported current users (20.5% vs. 11.6%); this suggests that about 9% had stopped using tobacco as at the time of survey, however, we do not know how many of these only experimented and how many actually progressed to regular use and quit. However, we know that youths often experiment with psychoactive substances [14]. More males used tobacco than females and this is consistent across various studies [4,6,8,15].

Introduction to tobacco use among current users were majorly by friends and this reflects the tremendous influence of peer pressure in initiating and sustaining youth behavior. Previous studies have also shown significant relationship between peer influence and smoking behavior among youths [16,17]. Major reasons for ever use and current use of tobacco were similar and include "to feel high and bold" and "because friends are using it". These major reasons emphasize the twin driving forces of quest for excitement and "belonging" in the life of adolescents.

A cross country comparison of youth tobacco use revealed that majority of current smokers purchased their cigarettes from a store [9]. This study revealed a similar pattern of buying spots in the motor parks being the greatest source of regular tobacco acquisition followed by non-commercial sources like friends and relatives. Although a group of researchers posited that proliferation of point-of sale restrictions may contribute to increase in non-commercial acquisition, it will be a wise decision to consider point-of-sale restriction within the context of a comprehensive youth programming approach [18].

About 40% of respondents believed that tobacco is not harmful to health. This is quite similar to the findings of a study done among senior secondary school students in an urban area where only 57% knew that smoking was harmful to their health [8]. Furthermore, our study shows that among current tobacco users, 84% did not know that tobacco use was harmful. This may suggests that current education programmes on tobacco use prevention in this LGA may be inadequate or inappropriate.

## Conclusion

Similar to a previous finding, this study found a high prevalence of life time tobacco use amongst adolescents who are poorly educated, out-of-school and live in rural areas [19]. Tobacco use prevalence amongst out-of school adolescents in this study was higher than the national average for in-school youths [20]. Our results also highlight the role of peer influence in starting tobacco use as well as easy access of out-of-school youths to tobacco products at motor parks in acquisition of tobacco. Our study only brings to mainstream research the peculiarity of this special group without providing further insight into the correlates of tobacco use. It would thus be essential that future researchers focus on in-depth analysis of tobacco use in this group to form the basis for recommending more targeted interventions by programme planners. Despite this limitation, the findings of this study suggests that a theory-based approach that seeks to understand out-of-school adolescents would be a good starting point for the design and implementation of an appropriate health education intervention targeted at communicating effectively the harmful nature of tobacco use to adolescents in this locality. A theory-based approach will assist us in understanding the logic of the behavior because perceptions, motivation, emotions, social environment and skills of the adolescent provide an explanatory logic which may guide the search for modifiable factors within this group of adolescents.

In addition, it might also be worthwhile to consider point-of-sale restrictions within the context of a comprehensive tobacco control policy to prevent access of adolescents to tobacco at youth aggregation areas.

## Competing interests

The authors declare that they have no competing interests.

## Authors' contributions

AOA and ETO conceptualized the study, AOA supervised the data collection. All authors participated in the analysis and interpretation of data. All authors participated in the report writing and read the completed article.
